# The physiology of survival: Breath‐hold shallow‐water diving

**DOI:** 10.1113/EP093322

**Published:** 2025-12-17

**Authors:** Andrew H. Baker, Ashley Jones, Adrian Mayhew, Carlene McAvoy, Ross Macleod, Hugh Montgomery, Craig Robertson, Jo Talbot, Mike Tipton

**Affiliations:** ^1^ Centre for Cardiovascular Sciences University of Edinburgh Edinburgh UK; ^2^ Swim England Loughborough UK; ^3^ Surf Lifesaving GB Exeter UK; ^4^ Royal Society for the Prevention of Accidents Birmingham UK; ^5^ National Water Safety Forum, RoSPA Birmingham UK; ^6^ Royal National Lifeboat Institution Poole UK; ^7^ Centre for Human Health and Performance University College London London UK; ^8^ Carnegie School of Sport Leeds Beckett University Leeds UK; ^9^ Royal Lifesaving Society Worcester UK; ^10^ Extreme Environments Laboratory University of Portsmouth Portsmouth UK

## 1 INTRODUCTION

Shallow‐water blackout and hypoxic blackout (SWB and HB; also referred to as ‘sudden underwater blackout syndrome’ and variations thereof; ILS, 2011) are characterized by the loss of consciousness in water. They can be fatal. They typically occur near the surface, but can occur at any depth and in many different circumstances and situations. SWB and HB often affect fit and healthy young people, but no one is safe. Understanding the risks and knowing how to avoid them when swimming underwater is thus essential in order to enjoy the water safely. The aim of this short paper is to help provide that understanding together with unified recommendations for prevention.

## 2 EXPERIENCE

SWB and HB have life‐or‐death consequences. Below are two personal experiences written to provide necessary context to the situations in which SWB and HB can arise, and the consequences and impact of it happening.

### 2.1 By Hugh Montgomery relating to his son Oscar

It was 27 May 2020, and the first day on which the lockdown lifted to allow day trips.

Oscar, my son, was 17 years old. Extraordinarily fit, he was a keen and talented rugby player. But his passion was the sea. Like me, he had been swimming from the age of 3, first used a mask and snorkel aged 4, and was a competent spear fisher at 12. By 16, every available recreational moment (even over Christmas) was spent in the sea, no matter how cold, rough or stormy.

The day was hot and sunny, the sea glass‐clear and calm. Oscar entered the water at ∼10.00 h. His camera suggests that he died not long afterwards. His body surfaced some weeks later.

I am to blame. I gave him his love of the sea and of breath‐holding to depth. I encouraged him to push himself, as I had always done. Nothing had ever gone wrong. For either of us.

But, looking back, he had last snorkelled nearly 9 months earlier; surgery (a rugby injury), then lockdown, had prevented him. He was even hungrier to go and fitter than ever (again, lockdown allowed). He would have wanted to push himself. And such clear water would have made the depths all the more enticing. He had had a new wetsuit delivered (lockdown prevented him getting to his standard gear) and would probably have misjudged weighting himself or the degree of buoyancy loss at depth.

Whatever. He died. And I miss him every day.

Pushing oneself physically and mentally should not be avoided. Doing so was part of Oscar's passion. This document seeks to present information that can help others enjoy the water as much as he did, whilst perhaps also helping to keep them that bit safer.

### 2.2 By Andrew Baker relating to his son George

We were on a family holiday in the South of France on 28 July 2022. George and I were in the communal pool, having a bit of fun seeing who could swim the furthest underwater without coming up for a breath.

We managed two attempts, going ∼25 m each time. Then I got out of the pool. George wanted to attempt the full length of the pool. He had another go, achieved 25 m or so again. He wanted one final attempt. After doing a little bit of hyperventilation, he went again. I was watching him closely, thankfully.

As George passed me, it looked like he then came up for air. But then he suddenly seemed to slow under the water. At this point, I immediately stood up and started to realize something was wrong as he became static underwater. I became increasingly anxious, so I ran down the side of the pool and very quickly entered the water. When I reached George, he was lifeless at the bottom of the pool, eyes open facing me. I got him out of the pool and onto the side in one move.

I immediately started chest compressions. As I was doing chest compressions, my daughter Serena had thankfully sensed something was wrong and ran over to where George and I were. On the way, she shouted for someone to call emergency services. Serena, now a qualified doctor, was a fifth‐year medical student at the time and was truly brilliant. She checked George's airway whilst I continued chest compressions, and then Serena also performed chest compressions. I cannot remember the exact timings. George did not show an immediate response, perhaps another minute passed. I think he had had ∼3 min without oxygen in total, and I felt as if the world was quickly collapsing.

Suddenly, some water started to dribble from his mouth, and he started to breathe slowly. He then started to make some horrific noises that will stay with me forever, I then thought he had serious brain damage at this time. However, things started to improve quickly, and within about the next 2 min, for the first time, I thought George might be okay. George was taken to the Nimes hospital, where he spent 4 days undergoing numerous tests to identify an underlying cause for his cardiac arrest. There was none. None of us had ever heard of SWB or knew of the risks George was taking. We were so, so lucky that the situation allowed a complete recovery for George. I cannot imagine life without George, but I think about that every single day. We went to the edge of the abyss, but luckily returned.

## 3 THE PHYSICS, THE PHYSIOLOGY AND THE PROBLEM

The key to avoiding a problem is to understand its evolution. In terms of SWB and HB, this means identifying the physical and physiological mechanisms by which they come about (i.e., ‘the cause of the cause of death’). There are many routes by which a breath‐hold diver can become unconscious when underwater [e.g., ‘cold shock’ (cardiac and respiratory consequences) (Tipton, 1989), traumatic injury, seizure, swim failure or a fall in blood oxygen (‘hypoxia’; ILS, 2011)]. A variety of factors thus influence risk (e.g., recent experience, speed and route of entering the water, equipment worn, water temperature, duration of exposure and depth). Other factors (e.g., degree of physical fitness, acclimatization to breath‐holding, and psychological drive) also play a part. Here, we focus on otherwise fit and healthy individuals undertaking breath‐hold diving (BHD).

### 3.1 The physics

The pressure exerted by the weight of the 38.6 km of the Earth's atmosphere above us is 760 mmHg (101.3 kPa or 14.7 psi). Because water is a lot denser than air, 10 m of water provides one atmosphere of pressure. Therefore, at the surface of the water, the body is at one atmosphere. Swimming down increases pressure by a 10th of an atmosphere each metre, hence at 10 m the pressure exerted is two atmospheres (one of air and one of water). Doubling the atmospheric pressure on the chest also doubles the pressure of air in the lungs and halves the volume of the air contained within them (Boyle's law). On return to the surface, the pressure halves (from two to one atmosphere), and if nothing else has changed, the volume returns to its original level.

In the lung, there is a mixture of gases [primarily nitrogen (N_2_), oxygen (O_2_) and carbon dioxide (CO_2_)]. The pressure exerted by each of these gases is proportional to the percentage of volume occupied (Dalton's law): if 21% of the gas is oxygen at the surface, then it exerts 21% of one atmosphere of pressure (21% of 760 mmHg or 160 mmHg; this is its ‘partial pressure’, *P*). As pressure changes with depth, so too does that exerted by each gas, in proportion. Thus, at 10 m, the pressure of gas in the lungs is two atmospheres or 1520 mmHg, meaning that the pressure exerted by the 21% which is oxygen will be 319 mmHg. It is the difference between the partial pressure of gas in our lungs and in our blood that drives gas exchange in our bodies, with gases moving along the pressure gradient from higher‐ to lower‐pressure areas. Normally, for oxygen this is from the lungs ($P_{AO_{2}}$) to the circulating blood ($P_{aO_{2}}$) and for carbon dioxide from the circulating blood ($P_{\mathrm{aC}O_{2}}$) to the lungs ($P_{\mathrm{AC}O_{2}}$). These partial pressures of gases are particularly important in BHD.

### 3.2 The physiology: drive to breathe

The urge to breathe is driven principally by three factors: a raised $P_{\mathrm{aC}O_{2}}$ or lowered $P_{aO_{2}}$ in the blood, in addition to the duration of ‘inactivity’ in breathing muscles. The drive to breathe can also be influenced by other factors, including cold receptor afferent input when immersed in cold water (Tipton, 1989). The urge to breathe can also be overridden with increasing success by physiological and psychological adaptation (Barwood et al. 2024).

Breathing is controlled by the central chemoreceptors (in the medulla oblongata of the brain) and peripheral chemoreceptors (in the carotid arteries [‘carotid bodies’] and arch of the aorta). Both provide input to the nucleus tractus solitarii and ventrolateral medulla (medullary respiratory centres). The peripheral chemoreceptors do not provide input to the central chemoreceptors directly (although the central chemoreceptors are found in the medullary centres). These receptors respond to changes in hydrogen ion concentration ([H^+^]), which is largely proportional to the $P_{\mathrm{aC}O_{2}}$, with a rise in $P_{\mathrm{aC}O_{2}}$ causing a proportional rise in [H^+^], stimulating the chemoreceptors and thus the urge to breathe (or to return to the surface, if snorkelling or BHD). The central chemoreceptors are also sensitive to cerebrospinal fluid pH, whilst the peripheral chemoreceptors respond to both CO_2_/pH and O_2_, in addition to some metabolites and temperature.

Unlike many physiological responses, breathing (rate and depth) are also under voluntary control. People can hyperventilate when they want to or can, for a period, overcome the drive to breathe (breath‐hold).

### 3.3 The problem

The terms SWB and HB are often used interchangeably, but they are not synonymous, although both do result in a rapid fall in blood oxygen (hypoxia) supply to the brain that causes unconsciousness.

HB typically occurs whilst breath‐hold swimming (for instance, ‘doing lengths underwater’). Exercising muscle consumes oxygen. As the oxygen content of arterial blood falls, muscle extracts all the oxygen it can, leading to a steepening fall in venous oxygen content. As the heart pumps harder and faster, the speed of blood transit through the lungs increases, meaning that there is less time for it to pick up oxygen. As the oxygen content in the inflated lung reduces, there is less ‘driving pressure’ for oxygen to enter the circulation. Arterial blood oxygen content thus falls even more dramatically. This rapid fall means that the time between the initial drive to breathe and unconsciousness can be very short. If that drive is overridden voluntarily or by diminished brain function, unconsciousness and drowning can occur at any depth.

**FIGURE 1:**
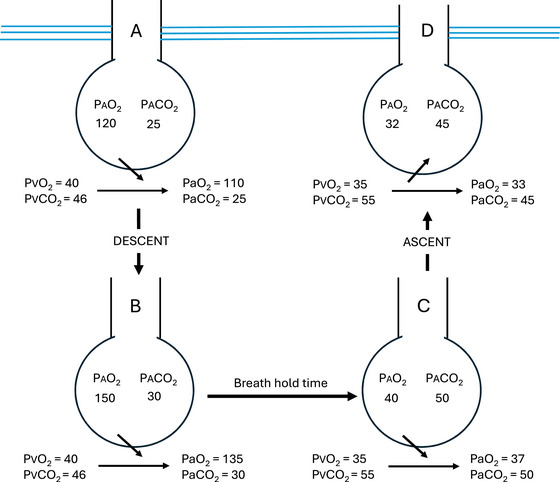
Mechanism of shallow‐water blackout during breath‐hold diving. (a) The diver hyperventilates before the dive, thereby reducing alveolar and arterial pressures of carbon dioxide ($P_{\mathrm{AC}O_{2}}$ and $P_{\mathrm{aC}O_{2}}$, respectively). (b) At depth, the $P_{\mathrm{AC}O_{2}}$ and $P_{\mathrm{aC}O_{2}}$ and the alveolar and arterial pressures of oxygen ($P_{AO_{2}}$ and $P_{aO_{2}}$, respectively) increase as the alveoli are compressed by hydrostatic pressure. (c) During breath‐holding, CO_2_ levels increase but can remain below the breath‐hold break point. Oxygen levels fall (note the decreased $P_{AO_{2}}$ and $P_{aO_{2}}$), and a hypoxic drive to breathe or unconsciousness may occur. (d) On ascent, particularly near the surface (greatest relative pressure change), $P_{AO_{2}}$ and $P_{aO_{2}}$ decrease as pressure falls and the alveoli re‐expand. Oxygen leaves the pulmonary capillaries and enters the alveoli. This can result in rapid profound hypoxaemia, unconsciousness and drowning. All values are expressed in millimetres of mercury. $P_{vO_{2}}$ and $P_{\mathrm{vC}O_{2}}$ represent the partial pressures of O_2_ and CO_2_ in venous blood, respectively. (From Brown & Gaitanaru in Brubakk & Neuman, 2003, modified from Brown & Piantadosi, 1998, with permission).

SWB refers to loss of consciousness on ascent from a breath‐hold swim to depth (e.g., free‐diving and spear fishing). A similar process to that in HB may contribute to SWB, although exercise is not necessary for this to occur, and changes in pressure on the body with submersion play an important role. An increase in intrathoracic pressure when diving raises the partial pressure of CO_2_ in the alveoli of the lung ($P_{\mathrm{AC}O_{2}}$), driving diffusion of it into the blood. With time, CO_2_ produced by body metabolism diffuses into the alveoli. Given that no air is being exchanged with the atmosphere, the $P_{\mathrm{AC}O_{2}}$ slowly rises until it reaches equilibrium with the $P_{\mathrm{aC}O_{2}}$ (Figure 1). This can mean that it takes a long time for $P_{\mathrm{aC}O_{2}}$ to reach a level that stimulates a drive to breathe. This situation is made worse if there is hyperventilation prior to diving; this lowers both total body CO_2_ content and $P_{\mathrm{AC}O_{2}}$. Swimming when underwater consumes oxygen, the ‘supply’ of which is supplemented by that from the raised (by being at depth) alveolar partial pressure of O_2_ ($P_{AO_{2}}$). Thus, the drive to breathe, which comes from a low $P_{aO_{2}}$ is also delayed. Furthermore, deoxygenated blood also has an increased capacity for CO_2_ carriage (Haldane effect), limiting the increase in $P_{\mathrm{aC}O_{2}}$ and the drive to breathe. Some examples follow, each of which ends with loss of consciousness and commencement of the drowning process.

Firstly, let us imagine someone in a swimming pool who is determined to beat their record for distance swum underwater (1 m depth). Swimming underwater consumes oxygen rapidly, resulting in a drop in the oxygen content of the body (hypoxia) that can cause loss of consciousness if the drive to breathe is overridden or, eventually, dulled by the impact of extreme hypoxia on neuronal activity.

Secondly, let us imagine someone in a swimming pool who is determined to beat their record for distance swum underwater (1 m depth). They know that hyperventilation immediately before the underwater swim will extend their breath‐hold time. By hyperventilating, they empty CO_2_ from their blood and lungs, lowering $P_{\mathrm{AC}O_{2}}$ and $P_{\mathrm{aC}O_{2}}$ and delaying the urge to breathe and return to the surface. Swimming underwater consumes oxygen, resulting in a drop in the oxygen content of the body (hypoxia) that can cause loss of consciousness before a sufficient level of CO_2_ is re‐established to evoke the urge to breathe.

Thirdly, let us imagine someone who free‐dives to 10 m (two atmospheres). This causes $P_{AO_{2}}$ to double, driving oxygen into the blood. Our diver swims at depth, their muscles consuming oxygen all the time, meaning that $P_{aO_{2}}$ and $P_{AO_{2}}$ fall. The CO_2_ generated by the muscle work diffuses into the lungs until $P_{\mathrm{AC}O_{2}}$ matches $P_{\mathrm{aC}O_{2}}$, at which point $P_{\mathrm{aC}O_{2}}$ begins to rise to a point that stimulates the drive to breathe. They thus begin to swim to the surface. Between 10 m and the surface, $P_{AO_{2}}$ drops by half (from two to one atmosphere), which now might be lower than $P_{aO_{2}}$. Oxygen will now start dumping from the blood to the lungs. Near the surface, as the relative pressure change maximizes, $P_{aO_{2}}$ falls rapidly to a level at which the brain cannot function. Loss of consciousness (ascent blackout) or a seizure occurs. The free‐diver drowns. If this occurs at sufficient depth, lung volume may be so low that the diver may be negatively buoyant (or they may breathe out as they lose consciousness), and they sink to the bottom. Hyperventilation before diving introduces the additional problems described above in the second scenario (Figure 1).

Craig (1961) describes eight incidents where the above occurred and, in 1976, described 58 more (Craig, 1968). Edmonds and Walker (1999) suggested that 15 of 60 snorkelling deaths in Australia also occurred in this way. Those who died were all males and were usually <40 years of age (Bart & Lau, 2023). In many cases, a focus on achieving a goal (competing on breath‐hold time or performing a task underwater, such as spear fishing) played a part by altering the interpretation of, or overriding, the physiological urge to breathe (Craig, 1961; Craig & Babcock, 1962; Craig & Medd, 1968; Craig & Harley, 1968).

The effect of hydrostatic pressure on alveolar gases is usually seen with dives beyond a depth of 5 m. However, SWB and HB incidents are commonly reported in shallower dives, such as in swimming pools. Therefore, BHD depth cannot account for all the incidents of SWB and HB. Hyperventilation reduces CO_2_ levels in the body, and the resulting increase in serum pH (alkalosis) promotes the binding of calcium ions to proteins such as albumin, reducing the amount of free (unbound) calcium in the blood. Given that calcium is involved in both muscle contraction and neurotransmitter function (possibly including the oxygen receptors), this can result in reduced transmission of neurotransmitters, loss of motor control, and bilateral fine motor tremor with head bobbing.

Competitive free‐divers call the euphoric sensations, disinhibition and altered perception they experience at the breath‐hold breakpoint ‘Samba’ (Gibson et al., 1981). It is associated with arterial hypoxia and possibly also a reduction in cerebral metabolic rate.

**FIGURE 2:**
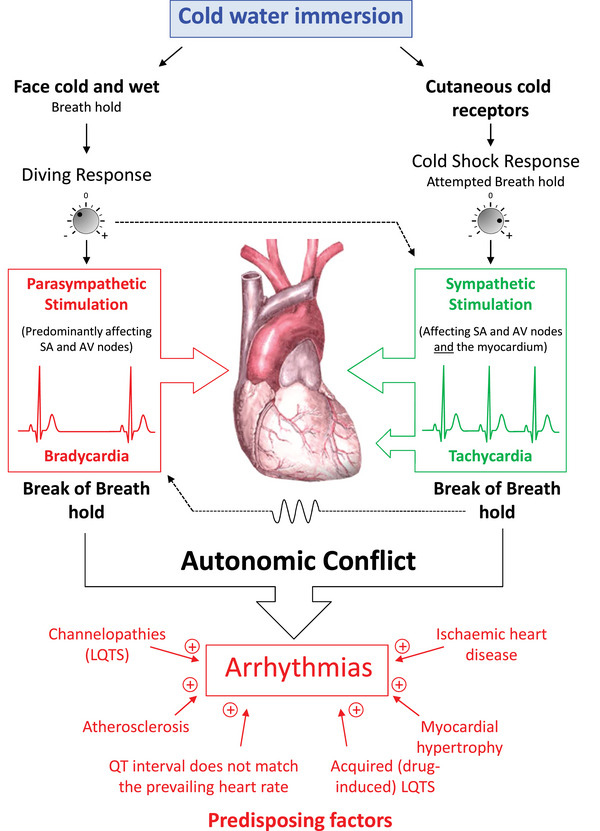
Cold‐water immersion activates two powerful responses: the diving response (upon facial immersion) and the cold shock response (on the activation of cutaneous cold receptors). The magnitudes of these responses can vary with a range of factors, including water temperature, clothing and habituation. Individually, the diving response triggers a parasympathetically driven bradycardia, whereas cold shock activates a sympathetically driven tachycardia. Together, these conflicting inputs to the heart can lead to arrhythmias, particularly at the break of breath‐hold, which increases parasympathetic tone that varies with respiration. The substrate for arrhythmias is enhanced by various predisposing factors, some of which (e.g., the failure of the QT interval to match the rapid and transient changes in heart rate) have chronic aspects but can also vary acutely (e.g., with drug consumption). Abbreviation: LQTS, long QT syndrome. From Shattock & Tipton (2012), with permission.

Hypoxia and hypocapnia can result in arrhythmias (Windsor et al., 2010). A low $P_{\mathrm{aC}O_{2}}$ can also reduce cerebral blood flow. It can drive a left shift in the oxyhaemoglobin dissociation curve, impairing tissue oxygen delivery. In addition, high vagal tone from the ‘diving response’ to face immersion in cold water can result in vagal arrest of the heart (Bayne & Wurzbacher, 1982; ILS, 2011). ‘Autonomic conflict’ (vagal stimulation as above, but with simultaneous sympathetic activation [e.g., ‘cold shock’]) can also cause lethal arrhythmias (Shattock & Tipton, 2012; Figure 2). Finally, breath‐holding at total lung capacity can, owing to intrathoracic compression, result in a fall in stroke volume and cardiac output when at the surface. This response is exacerbated with any form of glossopharyngeal insufflation (Ferrigno et al., 1986; Schipke et al., 2015).

## 4 PREVENTION, RESCUE AND TREATMENT

Common factors in the deaths of otherwise fit and healthy young breath‐hold divers and swimmers include two or more of the following:

- Prior hyperventilation (consciously or unconsciously [e.g., anxiety‐induced]).
- Extended breath‐holding.
- Submersion (body, including mouth and nose, below the surface of the fluid).
- Face‐only immersion.
- Level of exercise whilst breath‐holding.

The International Life Saving Federation (ILS, 2011) Medical Position Statement (MPS‐16) thus includes the following advice:

- Hyperventilation as a method of extending breath‐hold dive time should be actively discouraged.
- Any rescuer who observes an individual performing a BHD should have a low threshold for initiating rescue if the swimmer demonstrates signs of swim failure or ceases activity whilst submerged.
- In the case of unconsciousness or apparent cardiac arrest during a BHD, the resuscitation protocols appropriate to the level of training of the rescuer should be commenced. This may include ventilation with supplemental oxygen, chest compressions and the use of an automated external defibrillator. If advanced life support is available, consideration should be given to: ensuring a definitive airway; intravenous drug administration (including reversing metabolic disturbances, e.g., hypocalcaemia); and transfer to an appropriate critical care facility.

### 4.1 Prevention: location‐specific recommendations for avoiding SWB and HB

#### 4.1.1 Swimming pools

- Swimming pool lifeguards should discourage distance swimming underwater or remaining underwater for an extended period and warn pool users about the dangers associated with extended breath‐holding.
- Activities involving extended breath‐holding should be permitted only in a structured, organized session led by a competent instructor or coach, within a closed pool or closed area of the pool.
- Activities should be permitted only under the direct supervision of a competent instructor or coach following recognized safety protocols from a governing body for the particular activities.
- Careful consideration should be given to who provides lifesaving supervision. Swimming pool lifeguards may be unable to identify when a participant is having difficulty owing to the type of activity. Individual(s) providing supervision must be competent to:
  ○ Understand extended breath‐holding activities;
  ○ Identify when someone taking part in an extended breath‐holding activity requires assistance or is having difficulty;
  ○ Respond to those who require assistance or are in difficulty; and
  ○ Recover the casualty to the side of the pool.
- Many swimming clubs practice ‘lung buster’ drills, whereby the swimmer holds their breath for 50 m at a time and repeat multiple times. Often, these drills are used without the participant, parents or even some younger coaches being educated about the danger of SWB and HB. These drills should be avoided or very closely supervised.

#### 4.1.2 Elite coaching

Key considerations for coaches:

- Athletes must not hyperventilate before any underwater breath‐hold efforts.
- Compete for speed (high intensity), not distance, when carrying out underwater breath‐hold training.
- Athletes should never conduct breath‐hold training alone or in public swimming sessions. They should avoid breath‐holding at depth.
- The coach must inform the lifeguard of any planned breath‐hold training during a session. Supervision is always required by: (1) a coach with an understanding of breath‐hold training and when someone may be in difficulty; and (2) a lifeguard who is trained to recognize and respond appropriately to such incidents (e.g., Royal Life Saving Society, UK, and pool lifeguard) must be present.
- Care should be taken when an athlete is returning from illness.
- Never ignore a strong urge to breathe.

#### 4.1.3 Performance swimming specific

- Rest periods should be a minimum of 2–5 min between maximal breath‐hold efforts (maximal duration at maximal speed), and include consideration of observed recovery.
- For non‐maximal breath‐hold efforts (not maximal distance and/or maximal speed), athletes should have ≥30 s between breath‐holds, and include consideration of observed recovery.

#### 4.1.4 Artistic swimming specific

- Rest periods should be a minimum of 2–5 min between maximal breath‐hold efforts (maximal duration at maximal intensity), and include consideration of observed recovery.
- The rest periods should be tailored to the specific routine for breath‐hold exercises that are not maximal (not maximal duration and/or maximum intensity), and include consideration of observed recovery. If a new routine is introduced, appropriate progressions should be implemented to meet individual needs.

#### 4.1.5 Open water

- To mitigate any risk of SWB and HB in open coastal and inland water, swimmers must initially ensure that they understand the environmental risks associated with these waters. A risk assessment should be conducted. Mitigation of risks will include identifying a lifeguarded area and/or be under the supervision of a competent coach who is trained for such environments (Tipton et al., 2022).
- Visibility for any water‐based activity is always important. Being seen means that safety teams or coaching staff can quickly identify swimmers. Areas to consider include:
  ○ Wearing a high fluorescent hat (pink, orange or green);
  ○ Using a brightly coloured tow float;
  ○ Use of a wetsuit;
  ○ Carrying a pea‐less whistle on the wrist to attract attention;
  ○ Learning to float on your back; and
  ○ Learning to raise your hand to gain attention.
- Additional mitigation factors include swimming in a group and in an area where there is supervision. Those supervising should have appropriate rescue and first aid skills, including access to a defibrillator.
- BHD in open ocean water involves risks such as poor visibility, depth of water, and unforeseen change in the state of the ocean floor, such as a shifting sandbar. In inland waters, the risk can be the same, but instead of sandbars or coastal rock features, large stones or urban detritus are the major hazards that can be present (RoSPA, 2018).
- An activity such as BHD should be undertaken initially in a calm, risk‐assessed environment. It is essential that coaches teach swimmers not to hyperventilate prior to this type of activity. It is also critical that coaches and lifesavers are trained to understand the real risk associated with over‐extending breath‐holds and know what to look for and how to react. When such activity is taking place, the supervising person must be fully engaged in the safety of the person doing the activity and be prepared to act quickly to prevent dangerous behaviour or provide first aid care.
- Recommendations for BHD in open water are as follows:
  ○ Coaches should be fully competent to complete a full risk assessment of the site and match that with the person(s) undertaking the activity.
  ○ Ensure persons involved in the activity fully understand the types of risks in the environment in which they are active.
  ○ Wear personal protective equipment that is correct for the environment and that assists with visibility.
  ○ Enter the water slowly.
  ○ Agree on the signal for help (usually one hand waving).
  ○ Start with a gentle and slow process to assess an individual's capability and understanding of the activity.
  ○ Allow enough time between each activity for the person to recover fully.
  ○ Coaches should ensure that no hyperventilation or extended breath‐holding is taking place.
  ○ Ratios should always be 1:1 with a ‘buddy’, and coaches and lifesavers should be prepared to act.
  ○ Rescue equipment should be with lifeguards or competent lifesavers.
  ○ First aid equipment and a defibrillator should be easily accessible.

#### 4.1.6 Breath‐hold diving and snorkelling

- Be aware of the advice provided above and educate yourself about SWB and HB.
- Know the early signs of SWB and HB and do not attempt to override them.
- Undertake specialist training before diving, including free‐diving.
- Never dive or snorkel alone, although it is recognized that in some cases (e.g., spear fishing) you might end up some distance from others for safety reasons.

## AUTHOR CONTRIBUTIONS

Andrew H. Baker, Hugh Montgomery and Mike Tipton were involved in the conceptualization of the article. All authors contributed to the acquisition, analysis and interpretation of the work and drafting of the article and revising it for intellectual content. All authors approved the final version of the manuscript and agree to be accountable for all aspects of the work in ensuring that questions related to the accuracy or integrity of any part of the work are appropriately investigated and resolved. All persons designated as authors qualify for authorship, and all those who qualify for authorship are listed.

## ACKNOWLEGEMENTS

Thanks to Dr Paddy Morgan and Professor Mike Shattock for their comments on the manuscript.

## CONFLICT OF INTEREST

None declared.

## FUNDING INFORMATION

None.

## Data Availability

No data were generated or analysed for this study.
